# Interplay of stability and dynamics in the optimization of a highly proficient *de novo* enzyme

**DOI:** 10.64898/2026.05.29.728685

**Published:** 2026-09-10

**Authors:** Sagar Bhattacharya, Gessica M. Adornato, Yuda Chen, Xiao Huang, Wael E. Y. Mouloud, Hyunil Jo, Alexander N. Volkov, Ivan V. Korendovych, Yang Yang, David N. Beratan, Peng Liu, William F. DeGrado

## Abstract

The development of highly active *de novo* enzymes that catalyze new-to-nature reactions is becoming increasingly possible. Here, we identify the features underlying the catalytic efficiency of a highly proficient *de novo* enzyme, from the original computational design to an optimized catalyst obtained through two rounds of directed evolution. Computational, spectroscopic, and biochemical studies reveal successfully designed features, including precise alignment of catalytic residues, transition state stabilization, and environmental tuning. In the most evolved enzyme, binding of a transition state analog also led to widespread increases in backbone conformational stability throughout the protein, except within a helix near the active site entrance, where the introduction of Gly and Pro increased dynamics and catalytic activity. Thus, the entire protein contributes to catalysis in the most optimized enzyme. Also, the initial design considered only the transition state but not substrate binding, leading to a dynamic Michaelis complex prior to optimization. These studies show the multiple features that need to be optimized to achieve high activity in a designed enzyme.

## Full Text Availability

The license terms selected by the author(s) for this preprint version do not permit archiving in PMC. The full text is available from the preprint server.

